# *Healthcare* Reloaded: Announcing the New Aims and Scope of *Healthcare*

**DOI:** 10.3390/healthcare13182274

**Published:** 2025-09-11

**Authors:** Lorraine S. Evangelista

**Affiliations:** Louise Herrington School of Nursing, Baylor University, Dallas, TX 75246-1754, USA; l.evangelista@uci.edu

*Healthcare* (ISSN 2227-9032) [1] is among MDPI’s leading journals within the field of health research, systems, and care innovation. The healthcare sector is undergoing a global transformation due to an aging population, emerging technologies, evolving disease patterns, and an increased emphasis on equity and access. Our journal must evolve to reflect the significance of these changing developments.

Since its inception, *Healthcare* has served as a peer-reviewed, open-access forum for comprehensive research across all domains of health and medicine. In response to a changing landscape and in consultation with our editorial board and author community, we are proud to unveil our newly revised aims and scope [2]. This update reflects both the breadth of scholarship we currently support and the direction we aim to develop, deepening our engagement with clinical, community, technological, and policy-driven research that advances real-world outcomes.

Although the main concept of *Healthcare* focuses on improving health, patient care, and healthcare delivery, surrounding factors should not be overlooked, as they also influence healthcare workflows and outcomes. *Healthcare* seeks to encompass the roles of healthcare systems and organizations, including economic and management perspectives, while emphasizing the role of preventive medicine and digital technologies. These are to support sustainable and holistic healthcare approaches across diverse settings and populations. In addition, to improve clarity for authors and readers, the journal will not consider (i) instrument validation studies without demonstrated linkage to patient, population, or system outcomes, (ii) sports studies without clinical, public health, or health services relevance, or (iii) review protocols such as systematic or scoping reviews.

## 1. New Aims

*Healthcare* (ISSN 2227-9032) is an international, peer-reviewed, open access journal (free for readers) that publishes original theoretical and empirical research on all aspects of medicine and healthcare. The journal publishes Original Research Articles, Reviews, Case Reports, Technical Notes, Short Communications, etc. The journal covers all aspects of healthcare service, with an emphasis on healthcare management, health informatics, and health policy. *Healthcare* also publishes basic research relevant to clinical practice and patient care, encompassing prevention, diagnosis, treatment, and rehabilitation. Basic research studies of interest include those using (i) real-world clinical data or settings, (ii) measured patient, population, or health system outcomes, (iii) healthcare implementation insights, and/or (iv) health policy relevance. The audience of *Healthcare* includes all types of caregivers and healthcare professionals, such as nurses and physicians, as well as patients.

We encourage researchers to publish their experimental and theoretical results in as much detail as possible. For theoretical papers, full details of proofs must be provided so that the results can be checked; for experimental papers, full experimental details must be provided so that the results can be reproduced. Additionally, electronic files or software containing the full details of the calculations, experimental procedures, and other relevant information can be deposited along with the publication as “Supplementary Material”.

## 2. New Scope

This journal encompasses all topics relating to various aspects of medicine and healthcare research. Research fields of interest include, but are not limited to, the following:

**Nursing and Clinical Care**

- Acute Care;
- Long-Term Care;
- Specialty Care.

**Preventive Healthcare**

- Social Determinants of Health;
- Health-Lifestyle Behaviors;
- Screening Programs and Early Detection.

**Quality of Care**

- Medication Management;
- Patient Safety;
- Healthcare Delivery.

**Healthcare Technology**

- Artificial Intelligence;
- TeleHealth;
- Digital Technology;
- Health Informatics.

**Healthcare System, Management, and Policy**

- Healthcare Professionals;
- Healthcare Services;
- Healthcare Economics and Accessibility.

**Study Types That Will Not Be Considered for Publication by *Healthcare***

- Validation of instruments that are unrelated to healthcare outcomes;
- Sports studies without clinical, public health, or health services relevance;
- Protocols for review (systematic or scoping review).

## 3. New Sections

To ensure the content of the journal is aligned with the journal’s aims and scope, we have also restructured the sections of the journal into the following:

Artificial Intelligence in Healthcare;
Chronic Care;
Clinical Care;
Digital Health Technologies;
Healthcare and Sustainability;
Healthcare in Epidemics and Pandemics;
Healthcare Organizations, Systems, and Providers;
Healthcare Quality, Patient Safety, and Self-care Management;
Mental Health and Psychosocial Well-being;
Palliative Care;
Public Health and Preventive Medicine;
Women’s and Children’s Health.

Special Issues and papers will be reorganized to align with the new section structure.

## 4. Looking Ahead

The expanded scope of *Healthcare* demonstrates our commitment to publishing high-quality research on all aspects of health, healthcare delivery, and systems innovation. We remain committed to enhancing nursing and clinical care, with a focus on improving patient outcomes, facilitating seamless care transitions, and making the workforce more sustainable. As technology rapidly evolves within the healthcare sector, the journal consistently pursues innovative research on the topic. To highlight current changes within the healthcare field, we particularly welcome research that explores the potential applications of AI and machine learning in enhancing clinical diagnosis, decision-making, and administrative efficiency.

*Healthcare* presents itself as a dynamic journal that is ever-evolving, adaptive, and forward-thinking. It prioritizes scientific rigor, therapeutic relevance, and systemic transformation. We are pleased to operate as a fully open-access journal, providing authors globally with visibility and offering readers unrestricted access at no cost. We invite medical professionals, researchers, and influential thinkers from diverse disciplines to submit their most outstanding contributions to *Healthcare* and collaborate with us in advancing research that promotes equity, innovation, and excellence in global health.
